# Dynamic Echo Information Guides Flight in the Big Brown Bat

**DOI:** 10.3389/fnbeh.2016.00081

**Published:** 2016-04-25

**Authors:** Michaela Warnecke, Wu-Jung Lee, Anand Krishnan, Cynthia F. Moss

**Affiliations:** Comparative Neural Systems and Behavior Lab, Department of Psychological and Brain Sciences, Johns Hopkins University, BaltimoreMD, USA

**Keywords:** echolocation, sonar, animal flight, corridor, echo flow

## Abstract

Animals rely on sensory feedback from their environment to guide locomotion. For instance, visually guided animals use patterns of optic flow to control their velocity and to estimate their distance to objects (e.g., Srinivasan et al., 1991, 1996). In this study, we investigated how acoustic information guides locomotion of animals that use hearing as a primary sensory modality to orient and navigate in the dark, where visual information is unavailable. We studied flight and echolocation behaviors of big brown bats as they flew under infrared illumination through a corridor with walls constructed from a series of individual vertical wooden poles. The spacing between poles on opposite walls of the corridor was experimentally manipulated to create dense/sparse and balanced/imbalanced spatial structure. The bats’ flight trajectories and echolocation signals were recorded with high-speed infrared motion-capture cameras and ultrasound microphones, respectively. As bats flew through the corridor, successive biosonar emissions returned cascades of echoes from the walls of the corridor. The bats flew through the center of the corridor when the pole spacing on opposite walls was balanced and closer to the side with wider pole spacing when opposite walls had an imbalanced density. Moreover, bats produced shorter duration echolocation calls when they flew through corridors with smaller spacing between poles, suggesting that clutter density influences features of the bat’s sonar signals. Flight speed and echolocation call rate did not, however, vary with dense and sparse spacing between the poles forming the corridor walls. Overall, these data demonstrate that bats adapt their flight and echolocation behavior dynamically when flying through acoustically complex environments.

## Introduction

To navigate in the natural environment, an animal must estimate its relative distance to obstacles along its path in order to avoid collision and reach its goal. Previous research in flying animals, such as honeybees (Srinivasan et al., 1991, 1996; Baird et al., 2005), budgerigars (Bhagavatula et al., 2011) and *Drosophila* (Tammero and Dickinson, 2002) has provided evidence that these visually guided species rely on optic flow, the angular velocity of image motion across the animal’s eyes, to guide locomotion (Srinivasan et al., 1991). Optic flow provides continuous feedback to an animal about its relative velocity and distance to objects in its environment, and experimental manipulations of optic flow cues produce changes in animal flight trajectory and speed (Srinivasan et al., 1991, 1996; Baird et al., 2005, 2010; Dyhr and Higgins, 2010; Bhagavatula et al., 2011; Scholtyssek et al., 2014; Linander et al., 2016).

In the dark, optic flow cues become unreliable, and flying animals may need to rely on other strategies or sensory cues to move through a complex environment. For example, some bats have evolved an active sensing system – echolocation – to exploit echo-acoustic information to guide movements in the dark. Echolocating bats produce acoustic signals in the ultrasonic range and extract features of the environment from information carried by echo returns from surrounding objects. Some bat species use constant frequency (CF) signals, which are well suited to measure relative velocity (Schnitzler, 1973; Müller and Schnitzler, 1999), while other species rely exclusively on frequency modulated (FM) signals, which are well suited for spatial localization (Moss and Schnitzler, 1995; Simmons et al., 1995). Bats compute the distance to objects from the time delay between sonar emissions and echo returns (Simmons, 1973) and the angular offset of objects from inter-aural difference cues (Lawrence and Simmons, 1982; Simmons et al., 1983; Moss and Schnitzler, 1995). Populations of neurons in the bat auditory system show selective responses to 3D spatial acoustic information, i.e., distance (pulse-echo delay), azimuth and elevation, providing the neuro-computational substrate for dynamic sonar scene representation (reviewed in Suga, 1990; Dear et al., 1993; Ulanovsky and Moss, 2008). A recent neurophysiological study in the FM bat, *Phyllostomus discolor*, reports changes in echo-delay tuned neural responses to playbacks of pulses and echoes meant to simulate patterns of echo flow that a bat might receive as it flies past an obstacle (Bartenstein et al., 2014). At the behavioral level, changes in echo delay, inter-aural differences and Doppler cues must be integrated across time from successive vocalizations to render updated echo scenes. The intermittent sampling of spatial information through echolocation occurs at intervals spanning tens to hundreds of milliseconds and contrasts with the nearly continuous sampling of information through vision.

The big brown bat, *Eptesicus fuscus*, is a nocturnal mammal that flies at speeds between 2 and 6 m/s (Falk et al., 2014), while probing its environment with short, downward-sweeping FM biosonar sounds, which contain several harmonics in the range of 25–130 kHz (FM1: ∼65–25 kHz, FM2: ∼130–50 kHz) and last between 0.5 and 15 ms (Griffin, 1958; Simmons et al., 1979; Fenton and Bell, 1981; Surlykke and Moss, 2000). This bat dynamically decreases the duration and interval between sonar emissions as it approaches and intercepts prey (Simmons et al., 1979), and must rapidly process changing echo information to guide appropriate adjustments in flight and echolocation behavior (Surlykke and Moss, 2000; Simmons et al., 2001; Moss and Surlykke, 2010). Previous studies have shown that bats adapt echolocation call features in response to information extracted from echoes, which in turn influences the subsequent biosonar call design. For example, bats that produce CF signals adjust the frequency of their echolocation calls to compensate for Doppler shifts introduced by their own flight velocity (Schnitzler, 1968, 1973; Neuweiler et al., 1980). Both CF and FM insectivorous bats adapt the emission rate, duration, and bandwidth of their sonar signals, depending on changing distance to prey in both open and cluttered environments (Simmons et al., 1979, 2001; Surlykke and Moss, 2000; Schnitzler and Kalko, 2001; Surlykke et al., 2009). While performing obstacle avoidance and prey capture tasks that demand high spatial localization accuracy, FM bats adjust their call rate and produce sonar sound groups, clusters of echolocation calls with short intervals, flanked by calls at longer pulse intervals (Moss et al., 2006; Petrites et al., 2009; Kothari et al., 2014; Sändig et al., 2014). Therefore, echoes from objects in the bat’s environment directly affect the bat’s call timing, which in turn, directly influences the temporal patterning of echo returns. Laboratory research further demonstrates dynamic adjustments in flight speed, which are coordinated with adaptive echolocation behaviors (Petrites et al., 2009; Falk et al., 2014). For example, Petrites et al. (2009) show that the big brown bat decreases its call rate and flight speed with increasing clutter density, and they report an increase of sonar sound groups in high clutter conditions. Falk et al. (2014) further report shorter call durations in cluttered compared to open flight spaces. Overall, these results suggest that the big brown bat dynamically adapts its flight speed and biosonar call parameters when flying in cluttered environments.

**FIGURE 1:**
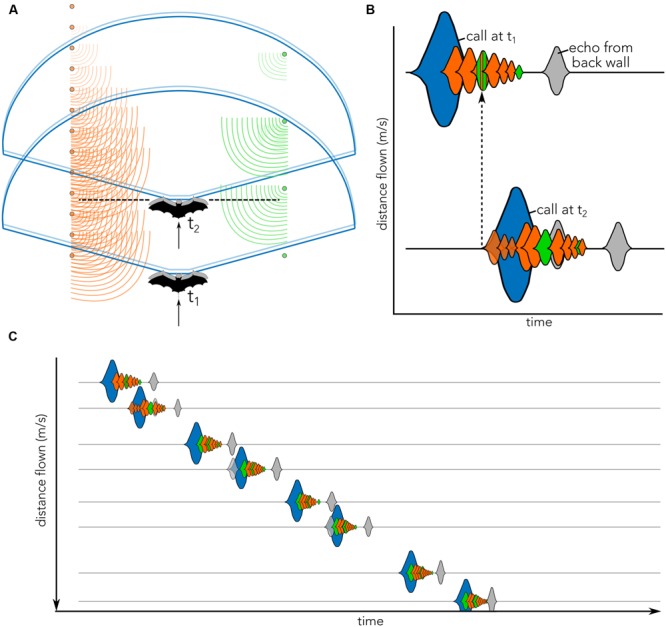
**Cartoon illustrating the concept of echo flow as used in the present article. (A)** Bat (black, *t*_1_) enters the tunnel and emits a vocalization (blue). Echoes return to the bat from the left (orange) and right (green) corridor sides. In the time that the bat emits the call (blue) it moves (gray), thereby slightly displacing the vocalization (light blue) and creating complex echo patterns. By the time the bat sends out its second call (black dashed line, *t*_2_), it has traveled further down the corridor, but some echoes from the previous call (*t*_1_) may still be returning to the bat. These echoes will overlap with the subsequent call emission (blue, *t*_2_) and echoes returning from call 2. **(B)** Biosonar vocalizations illustrated as sounds waveforms (blue) and echoes returning to bat from poles located at different distances (orange) from both corridor walls (orange vs. green) are shown across time (*x*-axis). Gray echo illustrates the echo from the wall at the end of the tunnel (see **Figure 2A**). The onset of call 2 (call at *t*_2_) occurs when echoes from call 1 (call at *t*_1_) are still arriving (dashed black arrow). The distance that the bat has flown in between two calls in schematically plotted on the (*y*-axis). Calls (blue) and echoes (orange/green/gray) are matched between panels **(A)** and **(B)**. Note that head and ear movements are not displayed and may further complicate echo patterns. **(C)** Schematic of waveforms across time (*x*-axis) as the bat flies down the corridor and emits successive vocalizations (blue). Pulse-echo overlap may occur (e.g., call 2, 4, and 6), complicating the spectral and temporal echo structures. The wall echo (gray) will increase in intensity and move closer to the time of vocalization as distance to the end of the corridor decreases. *Y*-axis plot distance flown between successive calls. Note that waveforms in **(B)** and **(C)** are cartoons illustrating the complex merging of the echoes returning to the bat; they do not represent how the bat perceives the flow of echoes.

Adjustments in bat flight and echolocation behaviors can be related to the echo-acoustic scenes the animal perceives via echolocation. Following each vocalization, a bat receives a cascade of echoes from objects ensonified by its sonar beam (**Figure 1A**). The horizontal beam of the big brown bat is spatially broad and spans approximately ±40-70°, depending on sound frequency (-6 dB beam width, Hartley and Suthers, 1989). Therefore, when the bat moves through its environment, the broad sonar beam will often ensonify not only prey, but also other objects. The bat’s analysis of its echo scene thus involves the integration and segregation of cascades of echoes arriving from different objects at different spatial locations and distances (Moss and Surlykke, 2010). This is a particularly challenging task, as echoes from closely spaced objects may overlap in time, creating complex interference patterns (Simmons et al., 1990; Bates et al., 2011).

Past studies of echolocation behavior in complex environments challenged the bat to maneuver around obstacles, which introduces uncontrolled echo-acoustic variables that are difficult to quantify across individual animals (e.g., Falk et al., 2014). In this study, we attempt to address this limitation by investigating the bat’s echolocation and flight behavior in experimentally controlled corridors, which constrain the animal’s flight trajectory and allow echo-acoustic information to be systematically varied. As the bat flies in the controlled corridor, it experiences differential changes of returning echo patterns over a series of echolocation calls. These echo cascades arrive at the ears of the moving bat and vary with the animal’s velocity, its head aim, and distance to objects in the environment (**Figure 1**). We here refer to these changes in echo patterns as “echo flow.” While the construction of our bat flight corridor was inspired by animal studies of visually guided flight, it is important to note that fundamental differences between vision and echolocation preclude direct comparisons between optic flow and echo flow.

In the present study, we investigated the effect of echo flow patterns on bat flight and biosonar behaviors. Echolocating big brown bats flew through a corridor with walls constructed from individual poles, mimicking rows of trees that a bat might encounter in its natural environment. This setup allowed us to manipulate the echo patterns of each corridor wall that each bat would receive as it flew. We hypothesized that the density of poles comprising each corridor wall would influence bat flight trajectory and echolocation behavior. Specifically, we predicted that bats would fly along the midline of the flight corridor with balanced pole-spacing on opposite walls, and that they would show wall-following behavior in conditions with imbalanced pole-spacing. We further hypothesized that the bats would adapt their call duration and use of sonar sound groups with the density of poles comprising the corridor walls. Specifically, we predicted that bats would shorten call duration and increase sound group production when they flew through corridors with walls comprised of more densely spaced poles.

## Materials and Methods

### Animals

**FIGURE 2:**
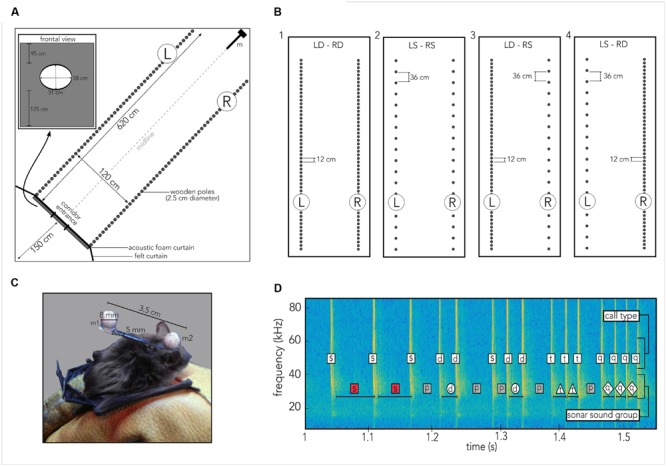
**Experimental procedures. (A)** Cartoon of the 6 m × 6 m × 2.5 m flight room, covered in acoustic foam, housing a corridor (620 cm × 120 cm) created from individual wooden poles (gray circles) so that the corridor’s left (L) and right (R) walls could be independently manipulated. Bats had been trained to fly through an elliptic hole (inset labeled “frontal view”) embedded in a rectangle of acoustic foam and black felt to prevent them from gaining echo information about the pole spacing prior to entering the corridor. The felt extended to the walls of the flight room (black line labeled felt curtain). Thirteen high-speed IR motion-tracking cameras were mounted within the tunnel (omitted for visual clarity) to capture the bat’s flight path, pole and microphone positions. Four ultrasonic microphones (m) were mounted vertically along the height of the room at the end of the tunnel. **(B)** Each of the seven bats flew at least ten trials in each of the following four different tunnel configurations. For each configuration “dense” refers to a 12 cm spacing of wooden poles, “sparse” refers to a 36 cm spacing of poles. (1) Left dense, right dense (LD-RD), (2) Left sparse, right sparse (LS-RS), (3) Left dense, right sparse (LD-RS), (4) Left sparse, right dense (LS-RD). **(C)** Photograph of the actual head marker, measuring 3.5 cm in length and 0.5 cm in width (original background grayed out) on the bat. Each reflective ball (m 1, m 2) to be tracked by a motion-tracking system is 8 mm in diameter. Head marker weighed 0.9 g total, making up about 5–7% of the animal’s body weight. Marker was attached using water-soluble theater glue. **(D)** Spectrogram illustrating sonar sound groups and call types as defined in the present study. Time series of calls is plotted as frequency (kHz, *y*-axis) across time (seconds, *x*-axis). For each call, the call type is indicated in the upper white box, while each sonar sound group type is indicated in the lower box. For sound groups, box shape and color correspond to marker shape and color in Supplementary Figure S2. Codes: s, single call/PI; d, doublet call/PI; t, triplet call/PI; q, quadruplet call/PI; p, pre-/post-sound group PI.

Seven wild-caught big brown bats, *E. fuscus* (three males and four females) were individually trained in an empty flight room to fly through a hole cut into a custom-built foam wall (**Figure 2A**, inset; see below). Bats were fed with mealworms (*Tenebrio molitor*) daily to maintain their individual weights between 13 and 16 g for the period of training and testing. The animals were housed in two group cages under reversed 12-h light/dark-cycle in a colony room kept at 24 to 28° C at 40 to 50% relative humidity. The experimental procedures were approved by the Johns Hopkins University Institutional Animal Care and Use Committee.

### Experimental Paradigm

Experiments were carried out in a custom-built carpeted flight room (6 m × 6 m × 2.5 m), with the walls and ceiling lined with acoustic foam (Sonex Classic, Sonex Acoustics, San Jose, USA) and shielded from outside electrical noise. Along the diagonal of the flight room a corridor was built from individually moveable wooden poles, 2.5 cm in diameter each that spanned from floor to ceiling (**Figure 2A**, gray circles). The corridor measured 6.2 m in length and 1.2 m in width. Each pole was individually set up and straightened by referencing to a Bosch self-leveling Line Laser (Robert Bosch Tool Corporation LLC., Michigan, USA). The left and right walls of the corridor were constructed with experimentally controlled spacing of the poles (**Figure 2**). Four conditions with different configurations of pole spacing on the left and right sides of the corridor were tested in the experiment (**Figure 2B**). At the opening to the corridor, a black felt curtain with an elliptic hole (31 cm × 38 cm; Rainbow Felt Black, Fabric.com, USA), attached to a large frame (251 cm × 120 cm) of acoustic foam prevented the bat from gaining information about the wall configurations until the animal began its flight through the corridor on each trial (see **Figure 2A**, black line labeled “felt curtain,” and gray bar and inset labeled “frontal view”).

Prior to each experiment, the designated bat was removed from its cage and allowed to fly for several minutes in an adjacent flight chamber. Once the bat was actively flying, water-soluble glue (Grimas Mastix Water Soluble, Heemstede, Holland) was used to attach a custom-built head marker to the bat’s head (**Figure 2C**). The head marker was 3.5 cm in length and 5 mm in width. Two reflective spheres (diameter: 8 mm) were glued to each end of the marker, so that one sphere was positioned between the bat’s ears (**Figure 2C**, m2), and the other was positioned on, but not glued to, the bat’s back (**Figure 2C**, m1). The total weight of the marker was 0.9 g, which corresponds to about 5–7% of body weight of individual bats. When the glue had dried and all recording systems had been configured, the experiment started. After collecting data over at least 10 trials, the head and body markers were carefully removed, and the animal was returned to its cage. Every day the seven different bats were tested in the same order and at the same time. For each trial three experimenters were present: the first experimenter released the bat from behind the curtain and otherwise stayed in that location. A second experimenter was responsible for catching the bat after a trial had ended and safely return it to the curtain-enclosed space. The “catcher” was otherwise waiting between the corner of the felt curtain and the flight room wall (**Figure 2**). The third experimenter recorded notes on every trial and triggered recording system for trial capture (see below).

**FIGURE 3:**
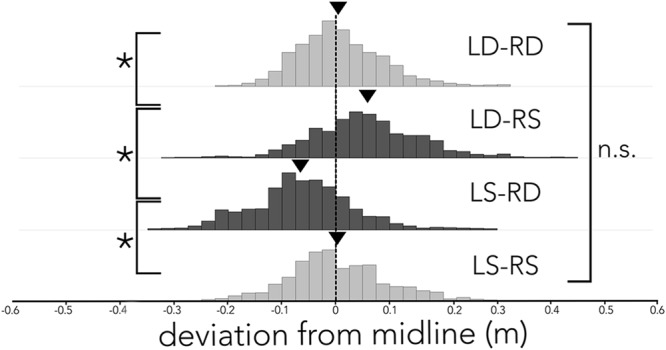
**Distribution of flight path deviation from the midline across conditions.** Histogram of the distribution of deviation (*x*-axis) from the midline (black dashed line) across all conditions (LD-RD, LD-RS, LS-RD, and LS-RS). Data are plotted in 2.5 cm bins, and balanced pole-spacing conditions (LD-RD and LS-RS) are illustrated in light gray, imbalanced pole–spacing conditions (LD-RS and LS-RD) are illustrated in dark gray. Average deviation from midline is indicated by a black triangle. Bats’ flight path differs significantly between acoustically unbalanced conditions (LS-DR and LD-SR), and unbalanced conditions differ significantly from balanced conditions. Balanced conditions (LD-RD and LS-RS) do not differ significantly.

**FIGURE 4:**
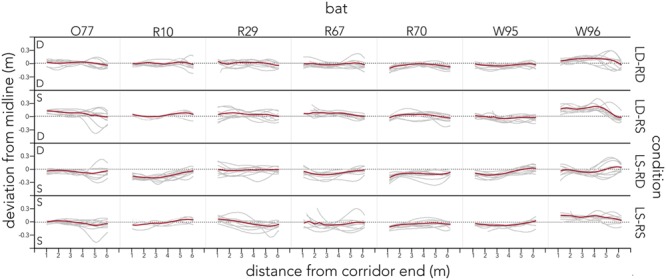
**Distribution of raw flight tracks across conditions and bats.** For each condition (right *y*-axis) raw flight tracks (gray) and their mean (red) are plotted as distance from the corridor end (lower *x*-axis). Tracks are plotted as deviation from the midline (black dotted line) along the left *y*-axis. The letter in the corner of each condition indicates the spacing of poles on that side of the condition. All data are plotted for each of the seven bats (upper *x*-axis). Bats’ flight paths steer away from densely spaced corridor walls and center otherwise.

Each bat navigated four different corridor configurations (**Figure 2B**) over a minimum of 10 trials. To study the bat’s flight and echolocation behavior in response to the density of clutter and the flow of echo information, we tested each animal in combinations of dense and sparse spacing of wooden poles. Due to the duration it took to prepare each corridor setup, a given condition was tested on a single day. For all conditions, dense spacing refers to a 12 cm gap between two poles, and sparse spacing refers to a 36 cm gap between two poles (see **Figure 2B**). Bats were released at ca. 1.5 to 1 m distance from the acoustic foam curtain, and they entered the corridor by flying through the entrance hole. Each of the seven bats navigated through a corridor whose two walls were comprised of densely spaced (**Figure 2B**, 1) or sparsely spaced (**Figure 2B**, 2) poles. These corridor wall configurations served as baseline conditions. To test the effect of imbalanced left/right echo patterns on behavior, each animal also flew through a corridor in which one wall was comprised of sparsely spaced poles (**Figure 2B**, 3R; 4L) and the other one of densely spaced poles (**Figure 2B**, 3L; 4R).

To restrict bats from using visual cues (Hope and Bhatnagar, 1979), all data collection was done in a dark room that was solely illuminated with dim infrared light for motion-tracking detection of the two reflective markers on the bat. Measurements of the light levels in the flight room at the beginning, middle and end of the corridor each revealed a light intensity of <10^-2^ lux. Measurements were done using a spectrophotometer (GS-1500, Gamma Scientific, San Diego, CA, USA) at experimental conditions.

### Data Recording

For each trial, synchronized audio and motion-tracking data of the flying bat were captured. Audio data were recorded using 4 ultrasonic microphones (D500X external microphone, Pettersson Elektronik Uppsala, Sweden) bandpassed between 10 and 100 kHz, mounted at the end of the corridor (**Figure 2**, m). Audio data were sampled at 250 kHz (NI PXI board 6143). The bat’s flight trajectory was acquired through 13 high-speed IR motion-capture cameras (Nexus, Vicon, Vicon Motion Systems Ltd., UK) mounted on the ceiling within the corridor. The motion-tracking system tracked the two reflective spheres attached to each bat at 300 frames per second. After all trials for the day were collected, the motion-tracking program also collected data on the position of the microphones, the location of the entrance hole, and the poles that made up the corridor walls. Every trial was manually triggered by an investigator after the bat had traversed the corridor at full length. Data acquired within 6 s prior to the trigger were stored for analysis.

Data from the motion-tracking system were processed oﬄine, and custom-written MATLAB programs (Mathworks, Natick, MA, USA) were used to digitally analyze the audio data and 3D flight tracks of the bat.

### Data Processing and Analysis

Motion-capture data were processed with custom-written MATLAB code to reconstruct 3D tracks of each bat’s navigational patterns on a given day. In subsequent processing, we used the 2D projection of the bat’s flight path onto the horizontal plane (floor) to compute its deviation from the midline of the corridor (**Figure 2A**, gray dashed line). Positive numbers indicate a deviation to the right side of the corridor, negative numbers indicate a deviation to the left side (see Results). Data points are calculated as distance from the end of the corridor, which has been defined as the plane created by the last poles on the left and right sides (**Figure 2A**, gray circles closest to ‘m’).

Echolocation calls produced by bats flying in the corridor were manually processed using an open-source MATLAB package that is archived on the GitHub software repository at: https://github.com/leewujung/call_marking_gui. We extracted the start and end times of each call that was emitted during the portion of the flight path that had been previously reconstructed. With these parameters we then calculated the call rate, pulse interval, and duration of each call.

For all variables of interest, data points at distances smaller than 1 m from the end of the corridor and greater than 6 m from the end of the corridor (the “start” of the corridor) were excluded. Only data collected from the middle portion (a total of 5 m) of the corridor were analyzed.

We used JMP to perform analyses with a mixed effects model on the relationship between experimental condition and parameters of interest (deviation from the midline, flight speed, call rate, call duration, pulse interval). A Tukey’s range test (HSD) was used for *post hoc* testing. For analysis of flight speed and sound parameters, we collapsed the two conditions that create an imbalanced flow of echoes (LS-RD, LD-RS) into one condition (S/D), as these conditions are essentially identical with regard to those variables. Unless otherwise noted, the mixed effects model analysis used condition (*N* = 4 for track data, *N* = 3 for all other data) as fixed effects and bat (*N* = 7) as random effects. *Z*-tests to test the deviation from the midline (zero-point) were done across condition and trials. Only trials in which the bat traversed at least AAA of the corridor were used for analysis: a total of 312 trials (LD-RD: *N* = 67, LD-RS: *N* = 64, LS-RD: *N* = 68, LS-RS: *N* = 62, LDS-RD: *N* = 51 trials) were analyzed.

## Results

Here we report how the spacing of poles along the corridor walls influenced the bat’s flight and echolocation behaviors. All flight data are plotted according to the bat’s deviation from the midline. Thus, negative numbers represent deviations to the left of the midline, whereas positive numbers represent deviations to right of the midline.

### Flight Tracks

**Figure 3** shows the bats’ average deviation from the midline (dashed black line) in the corridor across different conditions. When the corridor consisted of densely spaced poles on both sides (**Figure 3**, LD-RD), the bats centered their flight path and on average (black triangle) deviated 0.0075 m from the midline toward the right side. Similarly, when the corridor was comprised of sparsely spaced poles on both sides (**Figure 3**, LS-RS), the bats stayed close to the midline, deviating on average (black triangle) 0.0013 m from the midline. In both conditions, where the spacing of poles on either side of the corridor was imbalanced (**Figure 3**, LS-RD, LD-RS) the bats steered away from the wall with more densely spaced poles. Statistical analyses confirms a difference in the distributions of deviation from the midline across conditions. Using a Tukey’s HSD test we report that flight paths in LS-RD and LD-RS differ from baseline conditions (LD-RD, LS-RS) and from one another (*F*_3,18_ = 21.597, *p* < 0.0001; LD-RD: *M* = 0.0075 m, *SE* = 0.0004, LS-RS: *M* = 0.0013 m, *SE* = 0.0006, LD-RS: *M* = 0.055 m, *SE* = 0.0006, LS-RD: *M* = -0.0607, *SE* = 0.0005). The baseline conditions did not differ from one another. Importantly, flight paths in LS-RD and LD-RS also differ from zero (i.e., the midline of the corridor; *z*-test, LS-RD: z = -2.25, *p* = 0.012; LD-RS: *z* = 1.94, *p* = 0.026). In contrast, neither LD-RD nor LS-RS differ from zero (i.e., the midline of the corridor; *z*-test, LD-RD: *z* = 0.226, *p* = 0.82, LS-RS: z = -0.26, *p* = 0.79).

**Figure 4** plots the raw flight tracks (gray) and their mean (red) of each bat along the entire corridor for each condition.

### Flight Speed

**FIGURE 5:**
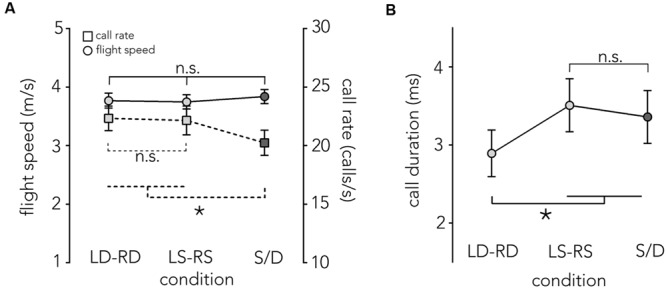
**Flight speed, call rate and call duration across conditions. (A)** Mean flight speed ±1 SE in m/s (left *y*-axis), is plotted across conditions (*x*-axis). When the animals fly though the different corridor manipulations (LD-RD, LS-RS, and S/D) flight speed (circles, solid line) is relatively stable at around 3.8 m/s with no significant differences across conditions. Also plotted is the mean call rate per second ±1 SE (right *y*-axis) across conditions (squares, dashed line). There are no significant differences in call rate in baseline conditions, though the difference between S/D and both baseline conditions is significant. **(B)** Mean call duration ± SE (*y*-axis) is plotted for each condition (*x*-axis). Bats use significantly shorter calls in the LD-RD condition compared to all other conditions (LS-RS, S/D). Asterisk indicates significance at *p* = 0.05 level.

**Figure 5A** compares the bats’ flight speed (circles and solid line) and their call rate (squares and dashed line, see below) across flight corridor configurations. On average, bats navigated all conditions at around 3.8 m/s. Flight speed did not differ across conditions (*F*_2,12_ = 1.77, *p* = 0.21; LD-RD: *M* = 3.76 m/s, *SE* = 0.0054, LS-RS: *M* = 3.74 m/s, *SE* = 0.006, S/D: *M* = 3.83 m/s, *SE* = 0.004).

### Echolocation Sampling

Previous research has shown that a change of flight speed in the echolocating bat is often accompanied by a reciprocal change in call rate (Petrites et al., 2009; Falk et al., 2014). We did not find a change in flight speed and also found that the call rates remained constant across baseline conditions, but showed a slight decrease in acoustically imbalanced conditions (S/D) (*F*_2,12_ = 5.7531, *p* = 0.017; LD-RD: *M* = 22.33 calls/s, *SE* = 0.077, LS-RS: *M* = 22.15 calls/s, *SE* = 0.096, S/D: *M* = 20.67 m/s, *SE* = 0.061, **Figure 5A**).

Overall, calls are shorter in the LD-RD condition (*M* = 2.89 ms, *SE* = 0.32) compared to the LS-RS condition (*M* = 3.5 ms, *SE* = 0.32) and S/D (3.36 ms, *SE* = 0.32) (*F*_2,12_ = 26.35, *p* < 0.001, **Figure 5B**).

**FIGURE 6:**
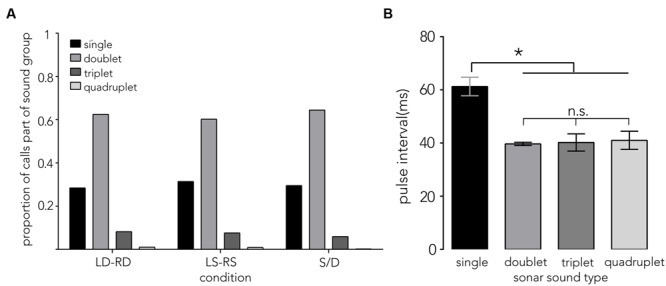
**Sonar sound groups and pulse interval distributions. (A)** Proportion of calls that are either single calls (single) or part of a sonar sound group (doublet, triplet, and quadruplet) is plotted on the *y*-axis across conditions (*x*-axis). Around 62% of all calls are doublets. **(B)** The mean pulse interval ±1 SE (PI, *y*-axis) that bats use when navigating the different corridor configurations show differences across sound group types (*x*-axis). Pulse intervals between single sounds (see red “s” in **Figure 2D**) are significantly longer, around 60 ms. By contrast, doublets, triplets and quadruplets share a shorter PI around 40 ms. Data are pooled across conditions, as no difference across conditions (LD-RD, LS-RS, and S/D) was found. Asterisk indicates significance at *p* = 0.05 level.

Of the 5,854 calls analyzed across conditions, 70.3% were part of a sound group (see **Figure 6A**, doublet, triplet and quadruplet), with calls creating a doublet call group making up the largest proportion (∼62%). The distribution of pulse intervals (PIs) was split into doublet sound groups (a single interval between 2 calls, **Figure 2D**, “d”), triplet sound groups (two intervals between three calls, **Figure 2D**, “t”), quadruplet sound groups (three intervals between four calls, **Figure 2D**, “q”) and single sound groups (intervals between single calls that did *not* immediately precede or follow another sound group (**Figure 2D**, red “s”, Supplementary Figure S2, red). Single pulse intervals that immediately preceded or followed a sound group (∼76%, **Figure 2D**, “p,” Supplementary Figure S2, black) were excluded from this analysis. The overall sound group distribution was analyzed using a random effects model with condition (*N* = 3) and sound group (*N* = 4) added as fixed effects, and bat (*N* = 7) added as random effects. **Figure 6B** illustrates the mean pulse intervals used within each type of sound group. We did not find an effect of condition (*F*_2,58_ = 0.2831, *p* = 0.75, LD-RD: *M* = 46.48 ms, *SE* = 3.78, LS-RS: *M* = 44.80 ms, *SE* = 3.69, S/D: *M* = 47.65 ms, *SE* = 3.78), but report that pulse intervals differ significantly across sound group types: pulse intervals between single sounds (red “s” in **Figure 2D**) are longer (*M* = 61.25 ms, *SE* = 3.74) compared to intervals between sounds that make up a sound group (*F*_3,59.49_ = 12.60, *p* < 0.0001, doublet: *M* = 39.68 ms, *SE* = 3.74, triplet: *M* = 40.20 ms, *SE* = 3.74, quadruplet: *M* = 44.10 ms, *SE* = 5.86).

## Discussion

Animals exploit a rich array of sensory cues to find their way in the environment, relying on vision, hearing, olfaction, somatosensation, infrared and magnetic sensing (Schone, 2014). Early work has suggested that animals relying primarily on vision for navigation, guide their movements through optic flow in order to measure their locomotion with respect to the objects in their surroundings (Gibson, 1958; Koenderink, 1986). Studies investigating the effect of optic flow on animal flight have provided evidence that several species (bees, budgerigars, and fish, among others) adapt their movement trajectories and speed in response to experimentally controlled optic flow cues (Srinivasan et al., 1991, 1996; Baird et al., 2005, 2010; Bhagavatula et al., 2011; Scholtyssek et al., 2014; Linander et al., 2016).

Here we investigated how an acoustically guided animal, the echolocating big brown bat, adapts its flight and sonar behavior in a restricted environment that returns dynamically changing patterns of echo flow. Inspired by paradigms used in optic flow research, we asked how the bat would change its flight trajectory, speed and biosonar behavior in a corridor with walls of differing echoic structure. In this corridor, each sonar emission returns a cascade of echoes from objects at different distances, some overlapping in time, which creates complex echo flow patterns (see **Figure 1**). These patterns change dynamically as the bat emits successive sounds in flight. It is noteworthy that acoustic flow in the corridor contrasts with optic flow along several dimensions. First, optic flow depends on relative movement between the animal and its environment, and provides a cue for relative distance. Echolocating bats, on the other hand, compute distance to objects directly from echo delay (Simmons, 1973). Second, a visual animal’s movement through the environment induces optic flow from light patterns present in the environment. In the case of echo flow, the dynamic features of echo cascades depend on the bat’s active and discrete sampling of sensory information from the environment. Third, optic flow is independent of the contrast or intensity of visual patterns (Srinivasan et al., 1991). By contrast, the structure and spatial configuration of corridor walls in our experiment directly influence the echo flow patterns the bat receives as it flies. Fourth, past research has shown that optic flow information can be experimentally manipulated or eliminated (e.g., Srinivasan et al., 1991; Scholtyssek et al., 2014). Echo flow information, however, cannot be removed from our flight corridor, whose walls return echoes that vary with the bat’s distance and directional aim of the sonar beam. These differences therefore limit direct comparisons between animal studies of optic flow and echo flow.

In accordance with our experimental predictions, the bats flew closer to the side with wider pole spacing when the corridor walls had different inter-pole spacing on the left and right sides (**Figure 3**, LS-RD and LD-RS). In conditions where the spacing was identical on both corridor walls, bats centered their flight paths, though they showed more variability in their flight trajectories along the corridor with wider inter-pole spacing (**Figure 3**, LS-RS vs. LD-RD), suggesting that reduced acoustic reflections from the walls influenced flight path planning. Our results show that bats deviate from the midline, away from the wall with dense pole spacing and toward the wall with sparse pole spacing. By contrast, bees continue to fly along the midline of a tunnel that displays different spatial frequencies of black and white vertical stripes on either side (Srinivasan et al., 1991). It is noteworthy that this independence of spatial frequency on the bee’s centering behavior holds only over a limited range of mismatch in the spatial frequency of patterns on opposite walls of the corridor. When the spatial frequency mismatch of vertical gratings on opposite walls is large, the bumblebees’ flight paths deviate toward the wall displaying higher spatial frequency grating patterns (Dyhr and Higgins, 2010). This finding differs from the flight path adaptations observed in our experiment, in which the echolocating bat veers away from the wall with higher density pole spacing. Note, however, that the spatial frequency mismatch of stimuli presented on opposite corridor walls in the study by Dyhr and Higgins (2010) cannot be directly compared to the differences in wall pole spacing in our study, as we manipulated the distance between poles, and not the width of poles on each side.

When bees and budgerigars are presented with corridor walls that display patterns of vertical stripes on one side and horizontal stripes on the other, animals adapt their flight paths to show wall-following behavior, as optic flow cues are absent from the wall displaying horizontal stripes (Srinivasan et al., 1991; Bhagavatula et al., 2011; Scholtyssek et al., 2014). As noted above, it is not possible to remove echo information from a structured environment, as all physical objects, including walls, return echoes, which are influenced by the bat’s distance to those objects as well as the directional aim of their sonar beam. In a previous study, bats abolished centering behavior in a flight corridor with horizontal and vertical poles on opposite walls (Supplementary Figure S1A), flying closer to the wall with horizontal poles (Supplementary Figure S1B) that return fewer echoes (Supplementary Figure S1C), LH-RV, LV-RH; Warnecke and Moss, 2015). While the overall bat flight paths in this earlier experiment mirror the flight adjustments observed in visually guided animals flying along corridors with vertical and horizontal stripes on opposite walls (e.g., Srinivasan et al., 1991; Bhagavatula et al., 2011; Scholtyssek et al., 2014), the findings of both research fields must be interpreted independently, as echo flow is not eliminated in horizontal pole condition.

Overall, bats produced shorter duration echolocation calls when flying through corridors with dense pole spacing (**Figure 5B**), which indicates that clutter influences sonar behavior. Specifically, shorter duration calls in highly cluttered conditions suggest a strategy that the bat might employ to reduce pulse-echo overlap from multiple, closely spaced reflecting surfaces (**Figures 1A,B**) (Petrites et al., 2009). Contrary to our experimental predictions, flight speed and echolocation call rate in baseline conditions did not vary with differences in the corridor wall pole spacing (**Figure 5A**) though on average, bats emitted fewer calls in corridors comprised of imbalanced echoic walls. The observed constant velocity suggests that the bat can adapt its flight behavior (deviation from the midline to steer away from dense pole spacing) without altering its velocity; this is consistent with studies on zebra fish swimming through manipulations of optic flow corridors (Scholtyssek et al., 2014).

Contrary to our prediction, the bats’ call rate did not increase from sparse (LS-RS) to dense (LD-RD) pole spacing conditions, and the use of sound groups (reciprocal of call rate), was consistently prevalent (**Figure 6A**, Supplementary Figure S2). In fact, bats emitted very similar proportions of sonar sound groups in all trials across conditions, producing doublets ∼62% of the time while flying through the corridor (**Figure 6A**, Supplementary Figure S2). The interval between single calls was significantly longer than the pulse intervals between clusters of sounds (**Figure 6B** single vs. doublet, triplet, and quadruplet). Past research suggests that sonar call groups may help the bat sharpen its representation of a complex environment (Kothari et al., 2014), and the consistent prevalence of sonar sound groups recorded from bats flying in the corridor (Supplementary Figure S2) suggests that this environment presents the bat with a challenging echo scene that requires frequent sonar probing. Supplementary Figure S2 illustrates typical and individual pulse interval patterns that each bat used when probing the corridor environment across conditions. It is noteworthy, that despite using different temporal patterning, the bats’ flight behavior is very similar across conditions (**Figure 4**).

Bats can use information carried by echoes to navigate narrow spaces, forage in cluttered environments, avoid objects in their path, and forage on the wing (Griffin, 1958; Schnitzler, 1973; Simmons and Kick, 1983). Similarly, visually guided animals use optic cues to maneuver in complex environments (e.g., Srinivasan et al., 1991; Baird and Dacke, 2012; Linander et al., 2016). The flight adaptations to echo flow patterns and optic flow cues suggest that common principles may underlie guidance of animal movement planning, even though the physics of light and sound render the stimuli distinctly different. While research has demonstrated that visual animals adapt their flight trajectories to balance optic flow, it is yet unclear whether bats adjust their flight paths to balance echo flow, or to simply steer away from the more echoic corridor wall. More detailed investigations into these phenomena may help elaborate on the computations involved in sensory-guided movement.

## Author Contributions

MW designed the experiment, collected, processed, and analyzed the data, and wrote the manuscript. W-JL wrote audio processing code and contributed to the writing of the manuscript. AK contributed to audio processing and the writing of the manuscript. CM contributed to the design of the experiment, discussion of data analysis and the writing of the manuscript.

## Conflict of Interest Statement

The authors declare that the research was conducted in the absence of any commercial or financial relationships that could be construed as a potential conflict of interest.
