# Supplementary material for: Dynamic Echo Information Guides Flight in the Big Brown Bat
Source: Front Behav Neurosci. 2016 Apr 25;10:81. doi: 10.3389/fnbeh.2016.00081 (PMC4843091; doi:10.3389/fnbeh.2016.00081)
Supplement: Supplementary file 1 [file Data_Sheet_1.DOCX]

Supplementary Material

**Dynamic echo flow guides flight in the big brown bat**

**Michaela Warnecke^*^, Wu-Jung Lee, Anand Krishnan and Cynthia F. Moss**

*** Correspondence:** Michaela Warnecke: [warnecke@jhu.edu](mailto:warnecke@jhu.edu)

# Supplementary Data 1

We provide supplementary data illustrating the results of an earlier experiment in which bats flew through a 375 cm long x 120 cm wide corridor built from 1-inch diameter PVC pipes, arranged either vertically or horizontally, and hung from the ceiling using fishing line (Power Pro Spectra, Braided fishing line, Power Pro, Irvine, CA). Each corridor wall was 120 cm high and hung from the ceiling, 120cm above the floor.. Six ultrasonic microphones were mounted at the end of the corridor and two additional microphones were located within the corridor to capture the bat’s echolocation behavior. Two high-speed IR cameras were mounted at the entrance of the corridor to record the 3D-flight path of each bat as it flew through the corridor. All trials were conducted under dim, long-wavelength (>650 nm) light to prevent the bats from using visual cues. In each trial, bats were released from the experimenter’s hand at the entrance to the corridor, flew through the setup and the previous 4 seconds of synchronized audio and video data were recorded after triggering when the bat had exited the corridor.

Different corridor wall manipulations were tested. In two baseline conditions, bats flew through either 1) two vertically hanging PVC pipe walls (14 cm spacing, LV-RV) or 2) two horizontally hanging PVC pipe walls (14 cm spacing, LH-RH). In experimental conditions, bats flew through corridors with horizontal and vertical pipes comprising opposite walls (LH-RV, LV-RH).

Data processing and analysis was the same as in the experiment reported in this manuscript.

The results show that bats’ flight behaviors are significantly different between the two baseline conditions (LH-RH: M = 0.04, SE = 0.017, LV-RV: M = 0.07, SE = 0.017) and the experimental conditions (LH-RV: M = -0.0862, LV-RH) (F_3,12_ = 34.48, *p < 0.0001*). Neither baseline condition is different from zero (z-test, LH-RH: z = 0.659, *p = 0.25*, LV-RV: z = 1.446, *p = 0.92*). While, as expected, LH-RV differs from zero (z-test, z = 2.575, *p = 0.005*), the broad distribution of flight paths in LV-RH does not render it significantly different from zero (z-test, z = -1.272, *p = 0.10*).

# Supplementary Data 2

We further provide a data figure to illustrate the different temporal patterns of echolocation calls made by individual bats as they traversed the corridor in each condition. Plotted is one sample trial per bat, per condition.

# Supplementary Figures

**Supplementary Figure 1.** Experimental paradigm and results of earlier echo flow experiment (Warnecke and Moss, 2015). **(A)** Schematic of the experimental setup illustrating a 375 cm long x 120 cm wide tunnel built from PVC pipes (yellow) that were combined into walls which could be individually hung from the ceiling. Walls were 120 cm in elevation. Individual poles were 14 cm apart in both vertical (here: left) and horizontal (here: right) walls. Bats’ flight patterns were recorded with two high-speed cameras and echolocation behavior was captured using 8 ultrasonic microphones (m). Bats were released from experimenter’s hand at the entrance to the corridor. Schematic depicts only one of 4 run conditions (LV-RH, see description above.) **(B)** Results of flight path deviations show that bats flew closer to the midline in baseline conditions (LH-RH, LV-RV), compared to experimental conditions (LV-RH, LH-RV). In experimental conditions bats deviated away from the vertically-spaced side and towards the horizontally-spaced side. **(C)** Spectrograms of echo measurements from the PVC corridor. Ultrasound signals were broadcast through an ultrasound loudspeaker (Ultra Sound Advice S56, London, UK) placed at the corridor entrance. The loudspeakeremitted a computer-generated hyperbolic frequency-modulated sweep, approximating the biological sonar signal of the big brown bat. Echoes were recorded with ultrasonic microphones (Ultra Sound Advice S56, London, UK) at the location of the loudspeaker. Spectrograms of echo returns in the LH-RH corridor (top) show fewer echoes (red) from the generated sweep (blue), compared to the imbalanced conditions (LH-RV, LV-RH, middle), and the LV-RV condition (bottom). Each spectrogram shows a pronounced wall echo (grey).

**Supplementary Figure 2.** Individual temporal patterning of bat echolocation calls. Each bat (top x-axis) shows different, but consistent, temporal patterning of pulse intervals (PI, left y-axis) across conditions (right y-axis) and corridor length (x-axis). Filled circles indicate single pulse intervals (refer to Fig. 2**(D)**), red circles indicate single PI, black circles indicate pre-/post- sound group PI (Fig. 2**(D)**, “p”). Pre-/post- pulse intervals are those that immediately precede or follow a sound group (either doublet, triplet or quadruplet, cf. manuscript). Open circles indicate doublet PI, triangles indicate triplet PI, diamonds indicate quadruplets.

**Reference**

Warnecke, E. and Moss, C.F. (2015) Navigating the world using echo flow patterns. *J. Acoust. Soc. Am. 137*, 2202 (2015); http://dx.doi.org/10.1121/1.4920008
