# Supplementary figures and images for: Dynamic Echo Information Guides Flight in the Big Brown Bat
Source: Front Behav Neurosci. 2016 Apr 25;10:81. doi: 10.3389/fnbeh.2016.00081 (PMC4843091; doi:10.3389/fnbeh.2016.00081)

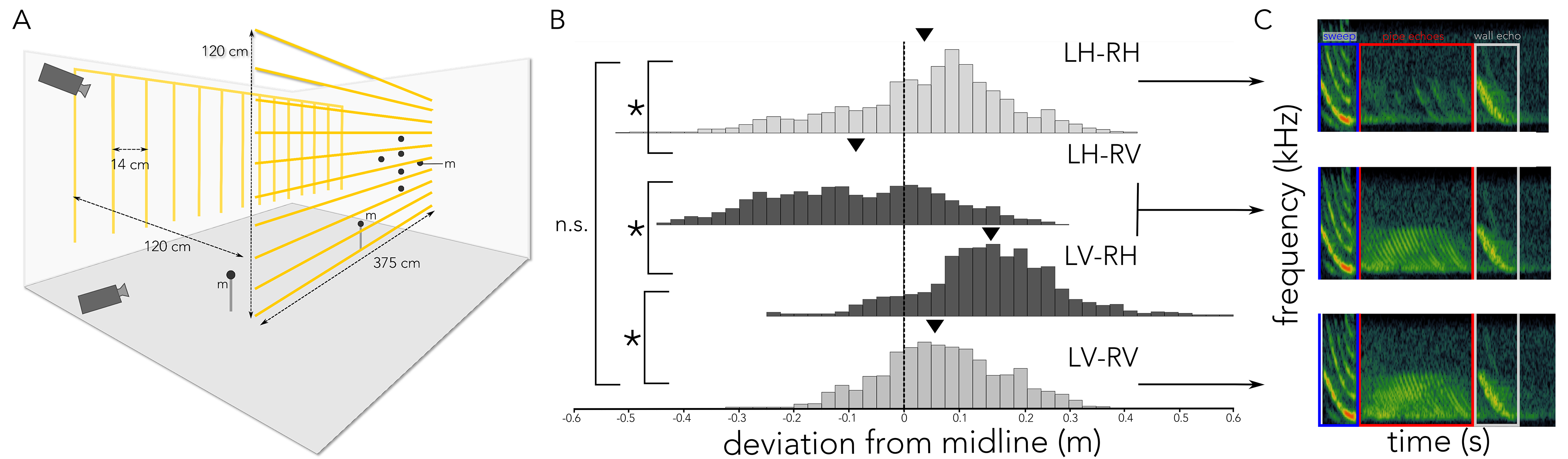

Supplement: Supplementary file 2 [file Image_1.TIF]

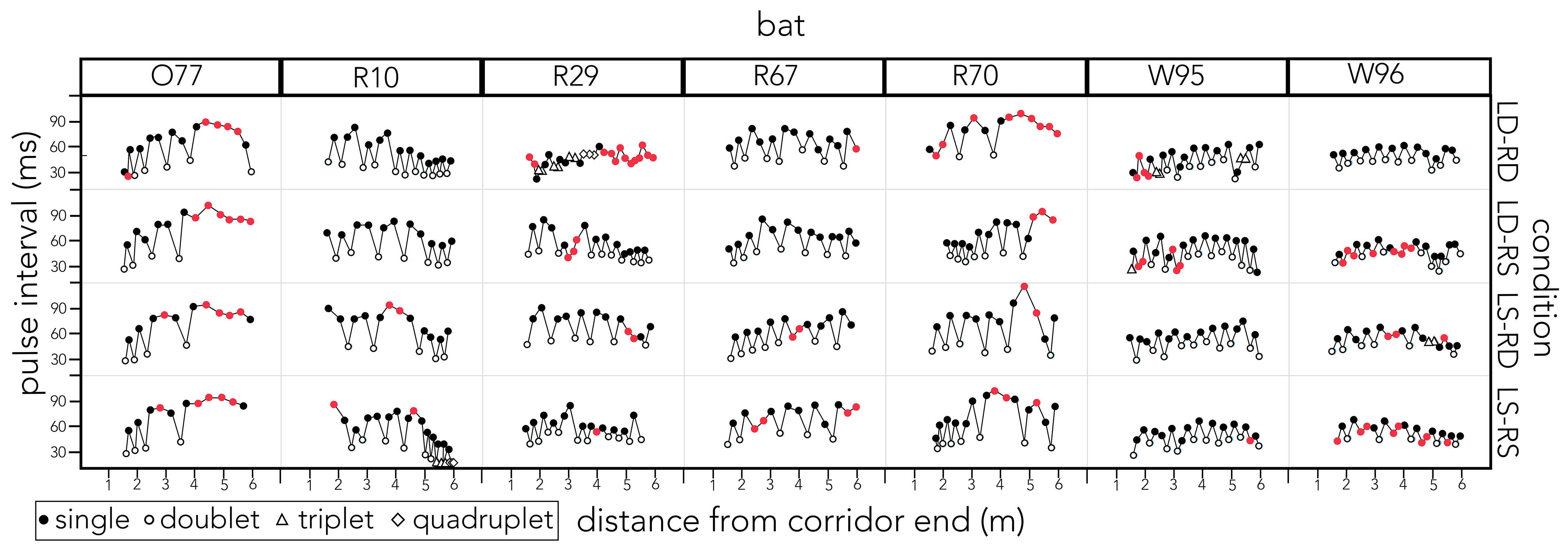

Supplement: Supplementary file 3 [file Image_2.TIF]
